# Endometriotic Peritoneal Fluid Stimulates Recruitment of CD4^+^CD25^high^FOXP3^+^ Treg Cells

**DOI:** 10.3390/jcm10173789

**Published:** 2021-08-25

**Authors:** Joanna Olkowska-Truchanowicz, Alicja Sztokfisz-Ignasiak, Aneta Zwierzchowska, Izabela Janiuk, Filip Dąbrowski, Grażyna Korczak-Kowalska, Ewa Barcz, Katarzyna Bocian, Jacek Malejczyk

**Affiliations:** 1 Department of Transplantology and Central Tissue Bank, Center of Biostructure Research, Medical University of Warsaw, 02-004 Warsaw, Poland; joanna.olkowska-truchanowicz@wum.edu.pl; 2 Department of Histology and Embryology, Center of Biostructure Research, Medical University of Warsaw, 02-004 Warsaw, Poland; ala.sztokfisz@gmail.com (A.S.-I.); izabela.janiuk@wum.edu.pl (I.J.); 3 1st Department of Obstetrics and Gynecology, Medical University of Warsaw, 02-015 Warsaw, Poland; teksanskamasakra@o2.pl (A.Z.); fil.dabrowski@gmail.com (F.D.); ewa.barcz@interia.pl (E.B.); 4 Department of Obstetrics and Gynecology, Multidisciplinary Hospital Warsaw-Miedzylesie, 04-749 Warsaw, Poland; 5 Department of Gynecology and Obstetrics, Medical University of Silesia, 40-055 Katowice, Poland; 6 Department of Immunology, Faculty of Biology, University of Warsaw, 02-096 Warsaw, Poland; gkorczak-k@biol.uw.edu.pl; 7 Laboratory of Experimental Immunology, Military Institute of Hygiene and Epidemiology, 01-163 Warsaw, Poland

**Keywords:** treg cells, Th17 cells, TGF-β, CCL20, cytokines, chemokines, endometriosis, peritoneal fluid

## Abstract

Endometriosis is a common gynecological disorder characterized by the presence of endometrial-like tissue outside the uterus. The disease is associated with disturbed local and systemic immunity. It has been reported that the proportion of CD4^+^CD25^high^FOXP3^+^ Treg cells may be significantly increased in the peritoneal fluid of patients with endometriosis. Therefore, the aim of our study was to investigate whether the proportions of Treg cells in the peritoneal cavity of patients with endometriosis are related to the chemotactic and stimulatory activity of the local peritoneal milieu. The peritoneal fluid was collected from 13 women with ovarian endometriosis and 12 control women without the disease. T cell populations were analyzed by flow cytometry, cytokines and chemokines were evaluated using the cytometric bead kit, and cell chemotaxis was studied by cell migration assay. We confirmed that the proportions of Treg cells are increased in the peritoneal fluid of women with endometriosis as compared to the control women. Endometriosis was also associated with elevated concentrations of IL-6, IL-10, and TGF-β1/2 as well as CCL20, CXCL8, CXCL9, and CXCL10. We did not reveal any changes in the proportion of peritoneal Th17 cells and concentrations of IL-17A. Peritoneal Treg cells positively correlated with concentrations of TGF-β, IL-10, and CCL20. Endometriotic peritoneal fluid stimulated chemotaxis of both CD4^+^ and Treg cells. This chemotactic activity positively correlated with concentrations of CCL20. CCL20 stimulated the migration of Treg cells, and the chemotactic activity of the endometriotic peritoneal fluid was inhibited by neutralizing anti-CCL20 antibodies. These results imply that increased proportions of the peritoneal Treg cells in women with endometriosis may result from attraction and activation by local chemokines and cytokines, especially CCL20 and TGF-β. Since Treg cells contribute to the immunopathogenesis of endometriosis, their chemotaxis and activation may be considered as a target for therapeutic intervention.

## 1. Introduction

Endometriosis is a common estrogen-dependent gynecological disorder that arises due to the presence of endometrial-like tissue (endometrial glands and stroma) located outside the uterine cavity, mainly on the pelvic viscera and/or ovaries [1,2,3]. The presence of ectopic endometrioid lesions is associated with chronic pelvic inflammation and the clinical manifestations of endometriosis include dysmenorrhea, dyspareunia, and/or chronic pelvic pain [4]. The disease may be also associated with subfertility and infertility [5,6]. Endometriosis affects about 10% of women in their reproductive age. It has a significant impact on patients’ life quality and constitutes a significant clinical and social problem.

Endometriosis appears to be a complex disorder of still poorly recognized etiopathogenesis that may involve the participation of different genetic, endocrine, environmental, and immune factors [1,7,8,9,10]. It is generally accepted that ectopic endometriotic lesions may develop from the endometrial cells shed during menstruation, which get into the peritoneal cavity in the course of a retrograde menstruation [2]. However, the mechanisms that facilitate survival and ectopic implantation and growth of endometrial cells are largely unknown. These phenomena may be at least partially explained by abrogated local estrogen production and progesterone resistance [9,10,11], as well as the resistance of endometriotic cells to apoptosis and their increased adhesiveness and invasiveness [2,12]. It is also possible that the survival of endometriotic cells is due to their impaired elimination by the cells of the local immune system, e.g., NK cells and macrophages [13].

There is a growing bulk of evidence that endometriosis is a chronic inflammatory condition that is associated with disturbed local and systemic immune reactions [14,15,16,17]. Endometriosis-related immune deviations may manifest by peritoneal infiltration with immune cells, activation of macrophages, abnormal lymphocyte responses, and impaired NK cell-mediated cytotoxicity as well as excessive accumulations of a bulk of proinflammatory and regulatory cytokines. Furthermore, endometriosis is associated with the elevated production of a variety of autoantibodies such as anti-nuclear, anti-phospholipid, and anti-endometrial antibodies [16,18,19,20]. These may strongly argue for endometriosis to be an autoimmune/autoinflammatory disorder. 

The etiopathogenesis of autoimmune/autoinflammatory disorders may be associated with the disturbed function of CD4^+^CD25^high^FOXP3^+^ regulatory T cells (Treg cells) [21,22]. Treg cells have been found to inhibit effector responses of T cells, macrophages, and NK cells. They also exert anti-inflammatory responses and inhibit the production of a variety of proinflammatory and stimulatory cytokines. Thus, Treg cells are considered as important mediators of immune tolerance. As such they appear to also be responsible for the repression of anti-tumor immunity and promotion of tumor growth [23]. The phenotype and function of Treg cells depends on high constitutive expression of CD25, a subunit of the IL-2 receptor, which is responsible for their development and progression, as well as the FOXP3 transcription factor which determines their development and suppressive activity [24,25].

Recently it has been reported that Treg cells may play a role in the immunopathogenesis of endometriosis [26,27]. Treg cells and increased *FOXP3* expression were reported in the eutopic and ectopic endometrium in women with endometriosis [28,29,30]. Furthermore, it has been found that the proportion of Treg cells may be significantly increased in the peritoneal fluid of patients with endometriosis [31,32,33,34,35]. Nevertheless, this observation has not been confirmed in some other studies [36,37,38]. Interestingly, an increased proportion of peritoneal fluid Treg cells may be associated with a decreased proportion of circulating peripheral blood Treg cells [31]. Increased numbers of Treg cells may have an impact on the local peritoneal immune responses including NK cells and may thus favor the survival of endometriotic cells and promote growth and invasion of endometriotic lesions. Changes in the proportions of Treg cells may also account for increased autoimmune phenomena, which frequently accompany endometriosis. Thus, Treg cells appear as important players in the immunopathogenesis of endometriosis and, therefore, may deserve special attention.

Endometriosis may also be associated with the increased numbers and upregulated activity of peritoneal Th17 cells [39,40,41]. Th17 cells are characterized by IL-17 production and are responsible for the stimulation of cellular immunity and inflammatory responses [42,43] that may be responsible for increased autoimmune and autoinflammatory reactions. Thus, their biological activity may counteract the suppressive functions of Treg cells. The role of Th17 cells in endometriosis remains, however, poorly recognized.

It is possible that immunopathogenic phenomena associated with endometriosis may depend on the Treg/Th17 balance [44]. However, the mechanisms responsible for the numbers of Treg and Th17 cells in the peritoneal fluid of women with endometriosis remain unclear. It is tempting to speculate that frequency and activity of both populations may be related to chemotactic and regulatory properties of the peritoneal fluid. Peritoneal milieu is known to be rich in a variety of cytokines/chemokines that may be potentially attractive for peripheral Treg and/or Th17 cells [14,45,46]. However, possible relationships and effects of the peritoneal milieu on Treg and Th17 cells have not been studied, so far. Therefore, the aim of the present study was to evaluate Treg and Th17 cells in the peritoneal fluid of women with and without endometriosis in relation to peritoneal concentrations of Treg- and Th17-related cytokines (IL-6, IL-10, IL-17A, and TGF-β) and chemokines (CCL2, CCL5, CXCL8, CXCL9, CXCL10, and CCL20). We also assessed the effect of the peritoneal fluid on their chemotaxis and activation.

## 2. Materials and Methods

### 2.1. Patients and Peritoneal Fluid Sample Collection

The study group included 13 women (median age 32 years, range 25–46) with laparoscopically and histopathologically confirmed endometriosis. All patients had ovarian endometriotic cysts and the stage of the disease was classified as moderate/severe (III/IV) according to the revised criteria of the American Society for Reproductive Medicine (rASRM) [47]. The control group consisted of 12 women (median age 31 years, range 19–46) without visible endometriosis foci, pelvic inflammation, or related pathology who underwent laparoscopic excision of ovarian dermoid cysts. The criteria of inclusion for both groups consisted of normal blood counts at admission to the hospital, regular menstrual cycles, and no history of previous pelvic surgery or chronic systemic disease. All women were in the mid-follicular phase (8–10 day) of the cycle at the time of laparoscopic examination. None of the women had undergone any hormonal or immunomodulatory therapy for at least six months prior to the study. The procedures were conducted according to the Helsinki Declaration of ethical principles and were approved by the Institutional Bioethical Review Board of the Medical University of Warsaw, Poland. All participants gave written informed consent to the study.

Peritoneal fluid was aspirated from the *cul de sac* at the beginning of the standard laparoscopic procedure under general anesthesia. Samples of peritoneal fluid contaminated with blood were excluded from the study. Peritoneal fluid samples were centrifuged at 400× *g* at 4 °C for 10 min and the cell-free supernatants were collected, aliquoted, and stored frozen at −80 °C until required for further evaluations. The leukocytes from the pellets were counted in a Bürker chamber, washed in ice-cold PBS, and suspended in an antibody staining buffer with a desired concentration for a further flow cytometry analysis. The yield of the peritoneal fluid and the peritoneal cells is shown in Table 1.

### 2.2. Flow Cytometry Analysis

For the flow cytometry analysis 0.5 × 10^6^ cells were labeled with 1 mg/mL of respective antibody for 30 min at 4 °C as described in detail previously [48]. For the identification and evaluation of Treg cells, freshly isolated peritoneal leukocytes or cultured CD4^+^ T cells were labeled with PerCP-conjugated anti-CD4 and APC-conjugated anti-CD25 monoclonal antibodies (both from BD Biosciences, San Jose, CA, USA). This was followed by a permeabilization-fixation procedure and intracellular staining of FOXP3 using the Phycoerythrin (PE) Anti-Human Foxp3 Staining Set (eBioscience Inc., San Diego, CA, USA) according to the instructions provided by the manufacturer. Identification and evaluation of peritoneal Th17 cells was performed by staining with the FITC-conjugated anti-CD161 monoclonal antibody (BD Biosciences) and Phycoerythrin (PE) Anti-Human ROR-γ antibody (eBioscience Inc.). For the evaluation of CD4^+^ T cell isolation efficiency (see below) the cells were labeled with FITC-conjugated anti-CD4 monoclonal antibodies (BD Biosciences). Nonspecific isotype IgG antibodies conjugated with the respective fluorochrome served as negative controls. 

**Figure 1:**
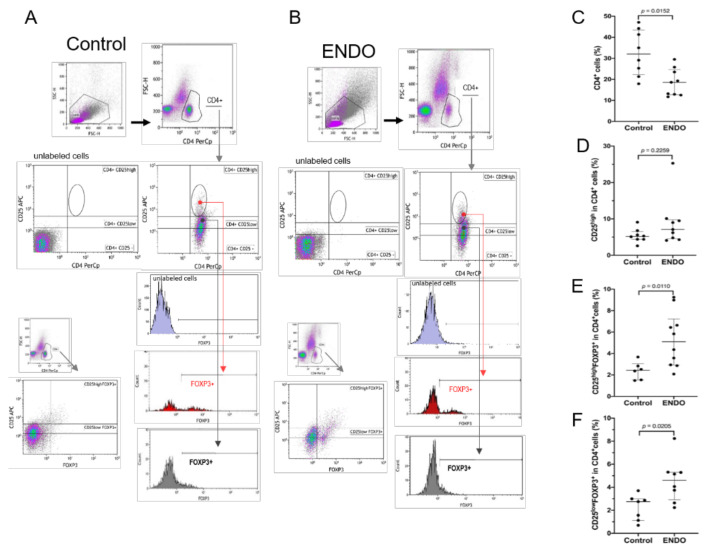
Flow cytometry gating strategy and analysis of the proportion of Treg cells in the peritoneal fluid from control women (Control) and patients with endometriosis (ENDO). Figure includes a representative flow cytometry analysis showing identification of the respective T cell populations in both (**A**) control and (**B**) endometriosis groups. The analysis included evaluation of the proportion of (**C**) CD4^+^, (**D**) CD4^+^CD25^high^, (**E**) CD4^+^CD25^high^FOXP3^+^, and (**F**) CD4^+^CD25^low^FOXP3^+^T cell populations as described in the Materials and Methods section. The results are shown as median and interquartile range. Statistical differences between groups were tested by two-tailed Mann–Whitney *U*-test.

**Figure 2:**
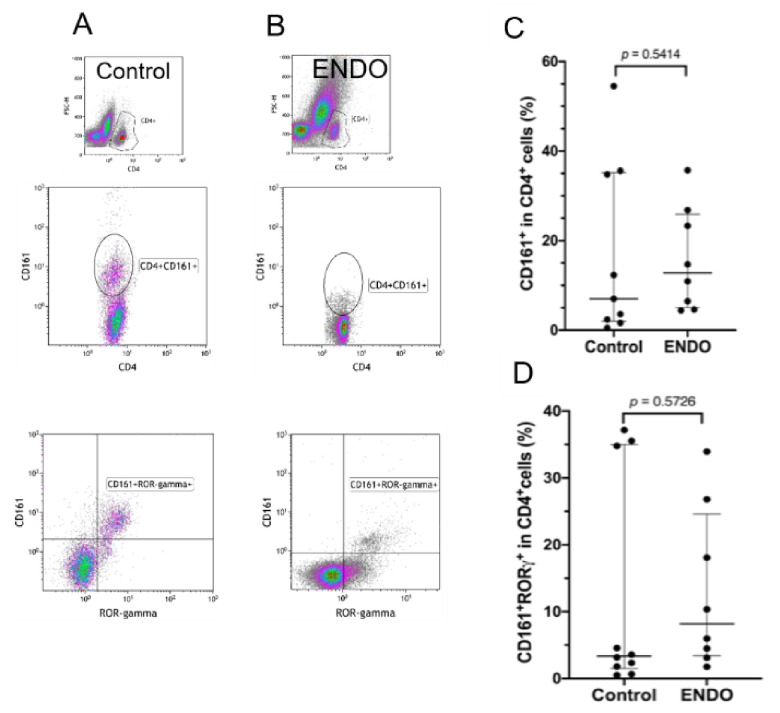
Flow cytometry gating strategy and analysis of the proportion of Th17 cells in the peritoneal fluid from control women (Control) and patients with endometriosis (ENDO). Figure includes a representative flow cytometry analysis showing identification of the respective T cell populations in both (**A**) control and (**B**) endometriosis groups. The analysis of the proportion of Th17 cells included evaluation of (**C**) CD161^+^ and (**D**) CD161^+^ROR-γ^+^ CD4^+^ T cell populations as described in the Materials and Methods section. The results are shown as median and interquartile range. Statistical differences between groups were tested by two-tailed Mann–Whitney *U*-test.

Samples were analyzed on the FACSCalibur using CellQuest / BD FACS Diva^TM^ software (BD Biosciences) and Kaluza Flow Cytometry Analysis software (Beckman Coulter, Brea, CA, USA). The cells were specifically analyzed by selective gating, based on the parameters of forward and side scatter as shown in Figure 1 and Figure 2. The results were based on the analysis of at least 100,000 cells and were shown as the percentage of positively labeled cells.

### 2.3. Cytokine and Chemokine Evaluation 

Concentrations of IL-6, IL-10, and IL-17A as well as CCL2, CCL5, CXCL8, CXCL9, and CXCL10 chemokines in the peritoneal fluids were measured using the BD™ Cytometric Bead Array (CBA) kits (BD Bioscience) according to the protocol provided by the manufacturer. Samples were evaluated using a FACSVerse flow cytometry with BD Suite software (BD Bioscience). The results were analyzed with FCAP Array software (BD Bioscience). Concentrations of TGF-β1, TGF-β2, and CCL20 were measured by specific Quantikine ELISA (R&D Systems, Minneapolis, MN, USA) using the FLUOstar Omega microplate reader (BMG Labtech, Offenburg, Germany).

### 2.4. Isolation of CD4^+^ and Treg Cells 

Peripheral blood mononuclear cells (PBMC) were isolated from the buffy coat by Histopaque (Sigma-Aldrich, St. Louis, MO, USA) density gradient centrifugation. Isolated cells were washed by centrifugation in PBS (Invitrogen, ThermoFisher Scientific, Waltham, MA, USA) at 400× *g* at 4 °C for 10 min. CD4^+^ T cells were isolated using the CD4^+^ Cell Isolation kit (Miltenyi Biotec, Bergisch Gladbach, Germany) and the Treg cell population was isolated using the Dynabeads™ Regulatory CD4^+^CD25^+^ T Cell kit (Invitrogen). Both populations were isolated according to the detailed protocols provided by the manufacturers. The purity of isolated cells was evaluated by flow cytometry as described above.

### 2.5. Chemotaxis Assays

Chemotaxis of isolated CD4^+^ T cells and Treg cells were evaluated by means of the CytoSelect^TM^ 24-Well Cell Migration assay (Cell Biolabs Inc., San Diego, CA, USA) in line with the protocol provided by the manufacturer. In brief, 5 × 10^4^ cells were resuspended in a serum-free RPMI 1640 medium (Invitrogen) and applied to the PET membrane inserts with 8 μm pore size in a 24-well plate (Cell Biolabs Inc.). Lower chambers were set up with investigated peritoneal fluid in RPMI 1640 at 1:1 ratio, CCL2 (50 ng/mL), or CCL20 (100 ng/mL) (both from R&D Systems). For investigation of the role of CCL20, peritoneal fluids were preincubated with specific neutralizing anti-CCL20 antibodies (R&D Systems) for 1 h at 4 °C. In all chemotaxis assays, the RPMI1640 medium with 0.5% BSA served as control. Following 18 h of incubation at 37 °C in 5%, CO_2_ cell migration was assessed fluorometrically using FLUOstar Omega microplate reader (BMG Labtech) and calculated according to the manufacturer’s formula.

### 2.6. Statistical Analyses

All statistical analyses including normality distribution testing and graphical presentations were performed using GraphPad Prism 8.2. 0 (GraphPad Software, San Diego, CA, USA). Statistical differences between the studied groups were tested by the two-tailed Mann–Whitney *U*-test or one-way analysis of variance (ANOVA) for independent or paired samples followed by a post hoc multiple comparison test. The relationships between the tested variables were analyzed with the use of a regression analysis or non-parametric Spearman rank-order method where applicable. *p* < 0.05 was considered statistically significant. The results are shown as median with interquartile range or mean ± SD.

## 3. Results

### 3.1. Analysis of the Peritoneal Treg and Th17 Cell Populations

The strategy and the results of the flow cytometry analysis of CD4^+^ T cell populations with the expression of the CD25 receptor and FOXP3 transcription factor in the peritoneal fluid from patients with endometriosis and control are shown in Figure 1. As can be seen, the proportions of the CD4^+^ T cells were significantly decreased in the endometriosis group compared to the control group (Table 1, Figure 1C). However, as seen in Table 1, a total count of CD4^+^ T cells was significantly increased which seems to be attributed to an increased count of total peritoneal cells. 

A further analysis showed that there were no significant differences between the endometriosis and control groups regarding the frequency of CD25^high^ cells among the CD4^+^ T cell population (Figure 1D). However, we found that the proportions of both CD25^low^FOXP3^+^ and CD25^high^FOXP3^+^ cells (Treg cells) within the CD4^+^ T cell population were significantly elevated in the peritoneal fluid from the endometriosis group as compared to healthy women (Figure 1E,F). 

A similar flow cytometry analysis of the proportions of CD161^+^ and CD161^+^ROR-γ^+^ cells in the CD4^+^ T cell population (Th17 cells) in the peritoneal fluid revealed no significant differences between the endometriosis and control groups (Figure 2).

### 3.2. Analysis of the Peritoneal Cytokine and Chemokine Concentrations

**Figure 3:**
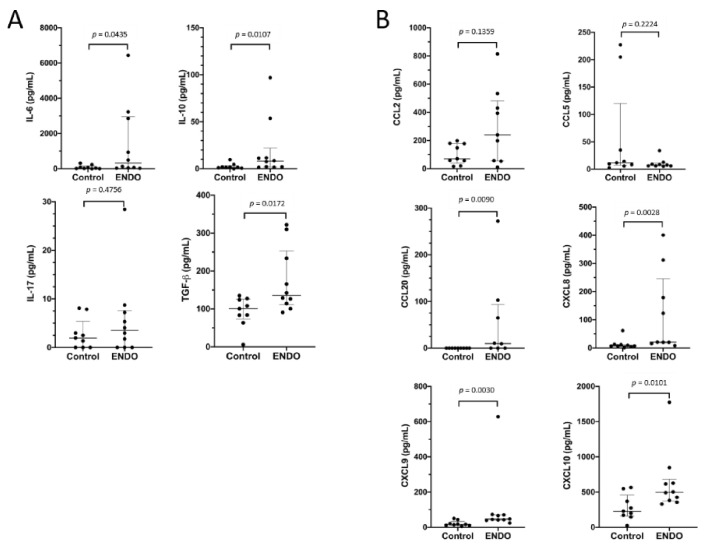
Concentrations of the immunoregulatory cytokines (**A**) and chemokines (**B**) related to generation and function as well as migration and activation of Treg and TH17 cells in the peritoneal fluid of control women (Control) and patients with endometriosis (ENDO). Peritoneal fluid concentrations of cytokines (IL-6, IL-10, IL-17A, and TGF-β1/2) and chemokines (CCL2, CCL5, CCL20, CXCL8, CXCL9, and CXCL10) were evaluated as described in the Materials and Methods section. The results are shown as median and interquartile range. Statistical differences between groups were tested by two-tailed Mann–Whitney *U*-test.

To ascertain whether the proportions of the peritoneal Treg and Th17 subpopulations may be associated with local immunoregulatory cytokines and chemokines we analyzed their concentrations in paired samples of the peritoneal fluid from patients with endometriosis and healthy women. The results of the evaluation of the peritoneal cytokines and chemokines that may be related to the generation and function of Treg and Th17 cells are shown in Figure 3. As can be seen, the concentrations of IL-6, IL-10, and TGF-β1/2 were found to be significantly higher in the peritoneal fluid of endometriosis patients as compared to the control group (Figure 3A). However, there were no differences between the endometriosis and control groups in the concentration of IL-17A in the peritoneal fluid. 

The evaluation of chemokines showed that the peritoneal fluid from patients with endometriosis displayed significantly higher concentrations of CCL20, CXCL8, CXCL9, and CXCL10 (Figure 3B). The levels of CCL2 and CCL5 in the endometriosis group did not differ compared to the control group.

### 3.3. Correlations Between the Peritoneal CD4^+^ T Cell Subpopulations and the Peritoneal Cytokines and Chemokines 

**Figure 4:**
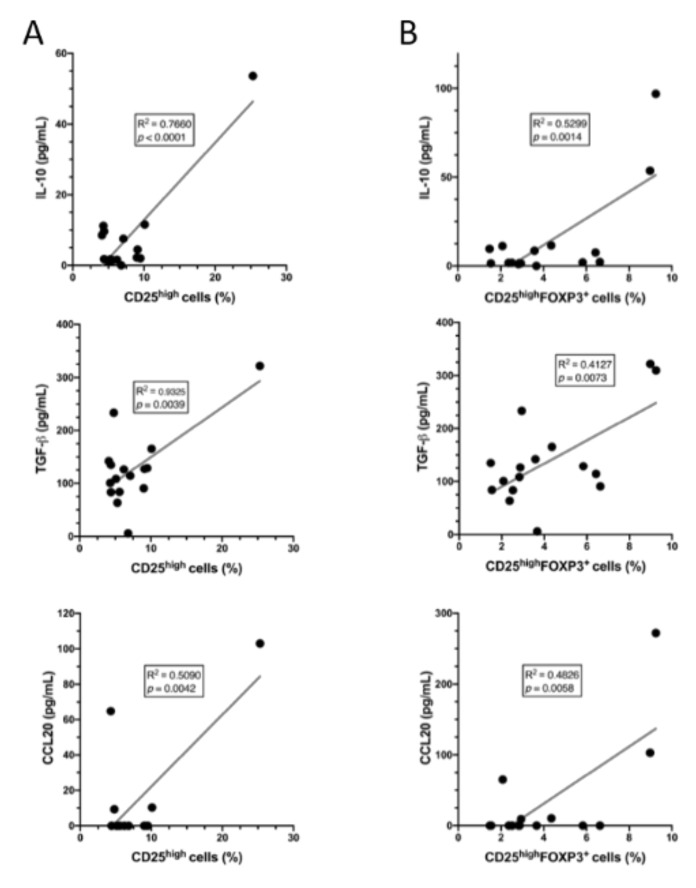
Correlations between the proportions of (**A**) CD25^high^ and (**B**) CD25^high^FOXP3^+^ CD4^+^ T cells and the concentrations of IL-10, TGF-β1/2, and CCL20 in the peritoneal fluid of women in the endometriosis and control groups. Correlations between the tested variables were computed by a linear regression analysis. Correlation coefficients (R^2^) and *p*-values are shown in the insets.

In order to reveal the correlations between the proportions of different peritoneal CD4^+^ T cell subpopulations and the concentrations of the peritoneal cytokines and chemokines, the results were subjected to a linear regression analysis. We found that the proportions of the general CD4^+^ T cell population did not correlate with any of the tested cytokines or chemokines (data not shown), whereas the proportions of both CD25^high^ and CD25^high^FOXP3^+^ cells significantly positively correlated with the concentrations of IL-10 and TGF-β1/2 (Figure 4). On the contrary, CD25^high^ and CD25^high^FOXP3^+^ cells did not correlate with either IL-17A or IL-6 concentrations (data not shown).

A significant positive association was found between the proportions of CD25^high^ and CD25^high^FOXP3^+^ cells and the concentrations of CCL20 (Figure 4); however, there was no correlation with the concentrations of CXCL9 or CXCL10 (data not shown). 

There was no correlation between the proportions of CD161^+^ and CD161^+^ROR-γ^+^ cells and the concentration of any tested cytokine or chemokine (data not shown). 

### 3.4. Characterization of the Chemotactic Activity of the Peritoneal Fluid and Identification of CCL20 as a Factor Responsible for Attraction of Treg Cells

**Figure 5:**
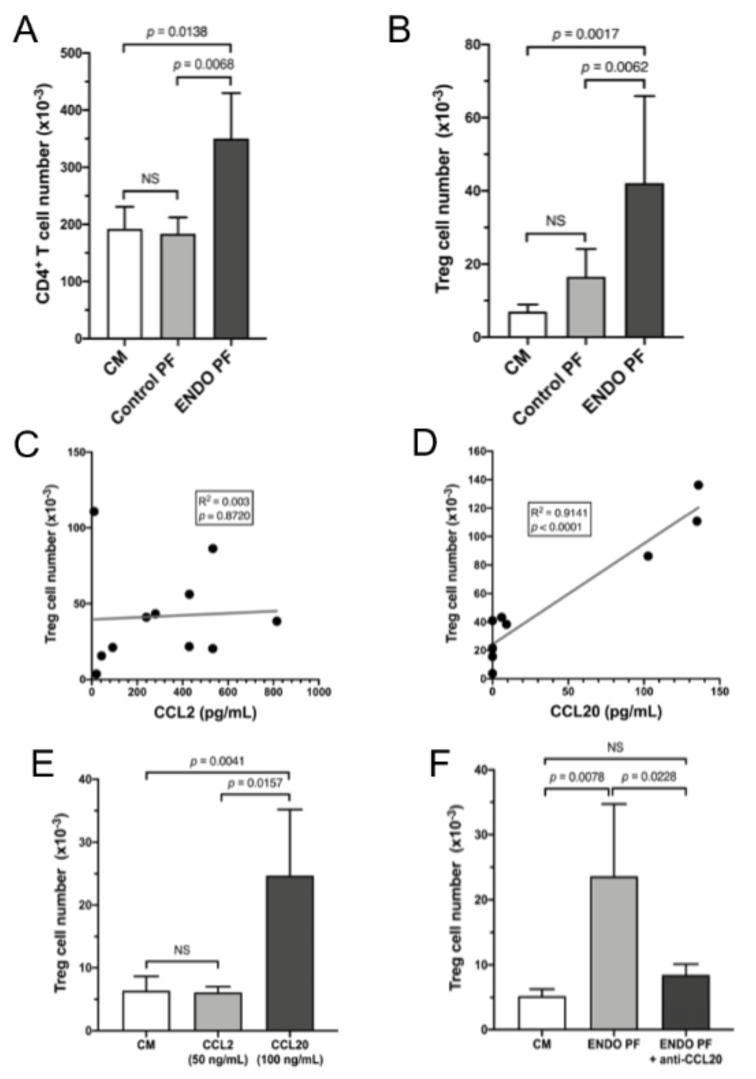
Demonstration of chemotactic activity of the peritoneal fluid from patients with endometriosis (ENDO PF) and control women (Control PF) for CD4^+^ and Treg cells and identification of CCL20 as a chemotactic factor responsible for attraction of Treg cells. Effect of control (*n* = 4) and endometriotic (*n* = 5) peritoneal fluid on chemotaxis of CD4^+^ T (**A**) and Treg cells (**B**). Correlations between the peritoneal fluid concentrations of CCL2 (**C**) and CCL20 (**D**) and the chemotaxis of Treg cells. (**E**) Effect of human recombinant CCL2 and CCL20 on the chemotaxis of isolated Treg cells (*n* = 4). (**F**) Effect of neutralizing anti-CCL20 antibody on the chemotactic activity of the peritoneal fluid from women with endometriosis (*n* = 4). Cell chemotaxis was evaluated as described in the Material and Methods section and is expressed as the number of migrating cells. Culture medium alone (CM) served as a negative control. Each bar represents mean ± SD. Statistical significance between groups was computed by the one-way analysis of variance (ANOVA) for independent samples followed by post hoc Tukey’s multiple comparison test. Correlations between the tested variables were computed by a linear regression analysis. Correlation coefficients (R^2^) and *p*-values are shown in the insets.

To reveal whether the peritoneal fluids from patients with endometriosis or the control women display a chemotactic activity toward CD4^+^ cells, and in particular Treg cells, isolated T cell subpopulations were investigated for migration in the presence of the tested fluids by means of the modified Boyden’s chamber assay. As shown in Figure 5A,B, both isolated CD4^+^ and Treg cells displayed significantly increased migration toward the medium containing samples of the peritoneal fluid from women with endometriosis as compared with peritoneal fluid from the control group and the medium alone. Compared to the medium alone, samples of the peritoneal fluid from the control women did not show any significant chemotactic activity.

The results of the linear regression analysis showed that there was no correlation between the chemotaxis of Treg cells and concentrations of CCL2 (Figure 5C) as well as CXCL9 or CXCL10 (data not shown). However, an increased migration of Treg cells was strongly correlated with increased concentrations of CCL20 (Figure 5D). These results strongly suggest that the migration of Treg cells may be mediated by CCL20. To confirm these observations, we performed experiments where the migration of Treg cells was tested in the presence of exogenous CCL2 or CCL20. As shown in Figure 5E, Treg cell chemotaxis was significantly stimulated by CCL20 whereas CCL2 did not exert any significant effect. The role of CCL20 as a chemokine responsible for the attraction of Treg cells by the peritoneal fluid of women with endometriosis was further confirmed in experiments with specific CCL20 neutralizing antibodies. As can be seen in Figure 5F, the addition of neutralizing anti-CCL20 antibodies resulted in a significant inhibition of the chemotactic activity of the peritoneal fluid from endometriosis patients almost to the level of migration observed in the culture medium control. 

## 4. Discussion

It has been reported that endometriosis is associated with increased proportions of the peritoneal Treg cells that may play a role in the immunopathogenesis of this disorder [26,27,29,31,34,35]. Nevertheless, the mechanism responsible for the accumulation of the peritoneal Treg cells in the course of endometriosis has remained obscure. The results of our present study provide new data showing that the proportions of the peritoneal Treg cells correlate with the concentrations of specific cytokines and chemokines. Furthermore, we found that the peritoneal fluid from women with endometriosis displays chemotactic activity toward Treg cells and that this activity at least partially depends on CCL20. These results strongly imply that the accumulation of the peritoneal Treg cells in the course of endometriosis appears be predominantly due to the chemotactic stimulatory effect of the local peritoneal milieu. 

Our present analysis of CD4^+^ T cells also revealed that their proportion may be decreased in the peritoneal fluid of women with endometriosis. However, a decreased proportion of CD4^+^ T cells was not associated with their decreased number or concentration in the peritoneal fluid and appears to be due to an increase in proportions of other infiltrating cells, as has been reported elsewhere [49].

Here we confirmed our previous results [31] as well as results of other investigators [34,35] that the proportions of the CD4^+^CD25^high^FOXP3^+^ Treg cells are significantly increased in the peritoneal fluid of women with endometriosis as compared with the control group. Unlike other researchers [40], we were unable to demonstrate statistically significant differences in the proportions of Th17 cells. However, it has been hypothesized that proportions of the peritoneal Treg and Th17 cells may depend on the stage of endometriosis [34]. Since the present study has been performed on patients with advanced stages of endometriosis, our results do not argue against the role of Th17 cells in the immunopathogenesis of this disorder. Thus, the significance of a Treg/Th17 balance in the course of endometriosis needs further elucidation.

Differentiation of Treg and Th17 cells depends on a variety of local regulatory cytokines including TGF-β1, IL-6, and IL-2 [50]. It is therefore plausible that the proportions of both T cell subpopulations may depend on the local milieu of the peritoneal fluid. Endometriosis is known to be associated with a plethora of various proinflammatory and immunoregulatory cytokines [14,45,51,52,53], and in the present study we confirmed that the peritoneal fluid of women with this disease contains increased concentrations of TGF-β1/2, IL-6, and IL-10. However, unlike others [41], we were unable to reveal increased concentrations of IL-17A that may reflect undisturbed proportions of Th17 cells. Proportions of Th17 cells also did not correlate with the levels of TGF-β1 and IL-6 which are considered as important factors involved in their development [50,54] thus further suggesting the limited role of Th17 cells in the pathogenesis of the advanced stages of endometriosis. 

On the other hand, we found that the proportions of peritoneal Treg cells significantly correlated with concentrations of both TGF-β1/2 and IL-10. This association is consistent with observations that TGF-β is an important factor involved in the differentiation and activation of Treg cells and has also been found to be a major regulatory factor released by these cells [55,56,57]. Increased concentrations of peritoneal TGF-β might also partially account for the increased proportion of peritoneal CD25^low^FOXP3^+^ (Figure 1F) which are likely inducible Treg cells differentiating from naïve CD4^+^ T cells [58]. On the other hand, the correlation between peritoneal Treg cells and IL-10 may be partially explained by the ability of these cells to produce and release this cytokine [59,60]. It should be stressed, however, that increased concentrations of IL-10 in the peritoneal fluid of women with endometriosis may not necessarily be a sole effect of increased proportions of Treg cells.

The proportions of different leukocyte populations in the peritoneal fluid may also depend on their attraction by local chemotactic factors. The peritoneal fluid of patients with endometriosis is rich in a variety of chemokines that account for peritoneal leukocyte infiltration in the course of the disease [46,61,62]. Some of them, e.g., CCL2, CCL5, and CXCL8 were even considered as possible markers of the disease [61]. However, in the present study we did not find that endometriosis is associated with the increased concentrations of the peritoneal CCL2 and CCL5. This inconsistency is difficult to explain; however, this might be due to the selection of patients with an advanced stage of the disease. Nevertheless, we confirmed our previous results [49,51] as well as other laboratories [61] that endometriosis is associated with elevated peritoneal CXCL8. While these chemokines may play a crucial part in peritoneal infiltration with monocytes/macrophages and some leukocytes, proportions of neither Treg nor Th17 cells correlated with the above-mentioned chemokines thus suggesting that these factors probably do not play a significant role in the local attraction of these T cell subpopulations. 

We also reported that the peritoneal fluid of patients with endometriosis showed significantly increased concentrations of CCL20, CXCL9, and CXCL10. Treg cells are endowed with receptors for both CCL20 (CCR6) and CCL9/10 (CXCR3) [63,64,65,66,67,68]. Accordingly, we found that their proportions correlated with peritoneal concentrations of CCL20. A similar correlation cannot be seen in the case of CCL9 and CCL10. These results strongly imply that the increased proportions of the peritoneal Treg cells in patients with endometriosis may result primarily from chemotaxis mediated by local CCL20. 

The chemotactic activity of the peritoneal fluid of women with endometriosis for isolated CD4^+^ and Treg cells was significantly higher when compared to the peritoneal fluid of the control subjects. A correlation analysis showed that the chemotaxis of Treg cells correlated with CCL20 concentration in the tested peritoneal fluid. A similar relationship for CCL2, CXCL9, and CXCL10 was not found. Further experiments revealed that the migration of Treg cells could be stimulated by CCL20 but not by CCL2, and the chemotactic activity of the endometriotic peritoneal fluid could be significantly inhibited by neutralizing anti-CCL20 antibodies. These results strongly imply that the increased proportions of the peritoneal Treg cells in women with endometriosis may be at least partially related to the increased concentrations of the peritoneal CCL20. It should be stressed, however, that Treg cells may also express other chemokine receptors such as CCR4, CCR8, or CCR10 [67,68,69] and the role of these other specific chemokines cannot be ruled out. Providing that the factors responsible for infiltration of the peritoneal cavity by Treg cells may constitute a potential therapeutic target for the treatment of endometriosis, possible involvement of other chemokines deserve further interest and extensive investigations.

CCL20 may be expressed in a variety of tissues and is involved in many pathological conditions including autoimmune disorders and cancer [70]; however, a mechanism responsible for an increased concentration of CCL20 in the peritoneal fluid of women with endometriosis remains to be elucidated. It is possible, however, that expression of this chemokine by endometriotic stromal and epithelial cells may be induced and increased by proinflammatory cytokines involved in endometriosis-associated inflammation, e.g., IL-1b, TNF, and IL-17 [71,72,73]. It is also noteworthy that IL-1b-mediated upregulation of CCL20 expression in endometriotic epithelial cells may be mediated by progesterone receptor B [73]. This information suggests that increased concentration of CCL20 in the peritoneal fluid of women with endometriosis is at least partially due to increased expression of this chemokine by endometriotic cells.

Interestingly, CCR6 is also expressed by Th17 cells [66,68] and, indeed, CCL20 may also play a role in chemotaxis and activation of these cells in the course of endometriosis [72]. Although we were unable to find any relationship between CD161^+^ROR-γ^+^ Th17 cells and the bulk of the studied cytokines and chemokines in the peritoneal fluid of women with advanced stages of endometriosis, similar relationships, e.g., at less advanced stages of the disease, cannot be excluded. Thus, further extensive studies are needed to reveal the mechanism(s) of the Treg/Th17 paradigm and its role in the immunopathogenesis of endometriosis.

## 5. Conclusions

In summary, the results of our present study elucidate the mechanism responsible for increased proportions of Treg cells in the peritoneal fluid of patients with endometriosis. We show that the increased proportions of Treg cells may be related to the local cytokine/chemokine milieu and to the local activity of the TGF-β as well as an increased chemotactic activity of the peritoneal fluid due to the increased concentrations of CCL20. It should be stressed, however, that the present study is limited to patients with ovarian endometriotic cysts at moderate/severe (III/IV) stages of endometriosis in the mid-follicular phase of the menstrual cycle and there is a need for further studies in less advanced stages of the disease and other stages of the menstrual cycle.

Providing that Treg cells play a role in the pathogenesis of endometriosis and may account for some endometriosis-associated autoimmune phenomena, our present observations revealing the putative mechanisms responsible for their recruitment and activation might be of clinical relevance and may define some novel targets for specific therapies.

## Figures and Tables

**Table 1:** Characteristics of the peritoneal fluid collected from control subjects and patients with endometriosis.

Peritoneal Fluid	Control Group (*n* = 12)	Endometriosis Group (*n* = 13)	*p*-Value
Peritoneal fluid volume (mL)	3.0 (0.5–13.0)	5.0 (2.0–12.5)	0.2073
Total cell number (×10^−6^)	1.85 (0.08–8.8)	5.4 (0.2–12.0)	0.1335
Cell concentration (×10^−6^/mL)	0.72 (0.08–1.7)	0.96 (0.1–2.93)	0.6724
CD4^+^ T cells (%)	32.05 (17.9–47.1)	18.60 (11.7–29.50)	0.015
Total number of CD4^+^ T cells (×10^−6^)	0.23 (0.06–0.75)	1.06 (0.23–1.61)	0.0079
CD4^+^ T cell concentration (×10^−6^/mL)	0.09 (0.02–0.37)	0.14 (0.05–0.54)	0.1457

Values are medians, range is given in parentheses. *p*-values were calculated by Mann–Whitney *U* test.

## Data Availability

Data are available from the authors upon request.
